# International Medical Congress

**Published:** 1886-09

**Authors:** Henry H. Smith, Richard J. Dunglison

**Affiliations:** Chairman; Secretary


### INTERNATIONAL MEDICAL CONGRESS.

CIRCULAR NO. 2.

### NINTH INTERNATIONAL MEDICAL CONGRESS.

The Ninth International Medical Congress will assemble in the city of Washington, the Capital of the United States, on Monday, September 5, 1887, at 12 o'clock noon, in accordance with the arrangements made at Copenhagen in August, 1884.

### PATRONS.

*The President of the United States.*—THE HON. GROVER CLEVELAND.

*The Secretary of State.*—THE HON. THOMAS F. BAYARD.

*The President of the Senate of the United States.*—THE HON. JOHN SHERMAN.

*The Speaker of the House of Representatives of the United States.*—THE HON. JOHN G. CARLISLE.

### PROPOSED OFFICERS OF THE CONGRESS.

*President.*—Nathan S. Davis, M. D., LL. D., Professor of the Principles and Practice of Medicine and of Clinical Medicine, Chicago Medical College, and of Mercy Hospital, Chicago, Illinois.

*Vice-Presidents, as far as appointed.*—Professor J. McCall Anderson, M. D., Glasgow, Scotland; Mr. Thomas Annandale, Professor of Clinical Surgery, Edinburgh University, Edinburgh, Scotland; Professor Dujardin-Beaumetz, M. D., Paris, France; Cuthbert Hilton Golding Bird, M. D., Professor of Physiology, Guy's Hospital, London, England; Professor Carl Braun, M. D., Vienna, Austria; William Brodie, M. D., Emeritus Professor of Principles and Practice of Medicine and Clinical Medicine, Detroit College of Medicine, Detroit, Michigan; W. W. Dawson, M. D., Professor of Surgery and Clinical Surgery, Medical College of Ohio, Cincinnati, Ohio; Thomas M. Dolan, M. D., Halifax, England; F. R. Frazer, M. D., Professor of Materia Medica and Therapeutics, University of Edinburgh, Edinburgh, Scotland; J. A. Grant, M. D., Ottawa, Canada; J. A. S. Grant, M. D., Cairo, Egypt; A. L. S. Gusserow, M. D., Professor of Obstetrics, Berlin, Prussia; Dr. Hans Ritter von Hebra, Vienna, Austria; Dr. E. Klein, London, England; Mons. Le Baron H. Larrey, Paris, France; Sir William MacCormac, Surgeon St. Thomas' Hospital, London, England; Mr. George B. Macleod, Professor of Surgery, Glasgow, Scotland; John S. McGrew, M. D., Honolulu, Hawaiian Islands; E. M. Moore, M. D., LL. D., Rochester, New York; Professor Von Monseil, Bonn, Prussia; Dr. Müller, Berlin, Prussia; William Murrell, M. D., London, England; Charles D. F. Phillips, M. D., M. R. C. S., late Lecturer on Materia Medica and Therapeutics, Westminster Hospital, London, England; Richard Quain, M. D., Professor of Anatomy, London, England; Tobias G. Richardson, M. D.,

Professor of General and Clinical Surgery, Medical Department, Tulane University, New Orleans, Louisiana; Mons. P. Ricord, Paris, France; Professor John Burdon Sanderson, M. D., London, England; Lewis A. Sayre, M. D., Professor of Orthopædic and Clinical Surgery, Bellevue Hospital Medical College, New York; Dr. Mariano Semmola, Professor of Experimental Therapeutics, University of Naples, Italy; Dr. Leopold Servais, Antwerp, Belgium; J. M. Toner, M. D., Washington, D. C.; Dr. P. G. Unna, Hamburgh, Germany; Professor F. Winckel, Dresden, Saxony; The President of the American Medical Association; The Surgeon-General of the United States Army; The Surgeon-General of the United States Navy.

*Secretary-General.*—John B. Hamilton, M.D., Supervising Surgeon-General of the United States Marine Hospital Service; Professor of Surgery, Georgetown University, D. C.; Professor of Surgery, Chicago Policlinic.

*Treasurer.*—E. S. F. Arnold, M.D., M.R.C.S., Newport, Rhode Island.

*Chairman of the Finance Committee.*—Richard J. Dunglison, M.D., Philadelphia, Pennsylvania.

*Chairman of the Executive Committee.*—Henry H. Smith, M.D., LL.D., Emeritus Professor of Surgery in the University of Pennsylvania, Philadelphia, Pennsylvania.

*Chairman of the Committee of Arrangements.*—A. Y. P. Garnett, M.D., Emeritus Professor of Clinical Medicine, Columbia University, Washington, D. C.

*Associate Secretaries of the Congress.*—William B. Atkinson, M.D., Philadelphia, Pennsylvania; G. B. Harrison, M.D., Washington, D. C.

The Congress will consist of such members of the Regular Medical Profession as shall have registered and taken out their ticket of admission, and of such other Scientific men as the Executive Committee of the Congress shall deem it desirable to admit. The books for the Registration of Members will be open from 9 A. M. to 5 P. M., on Thursday, September 1, 1887, and on each subsequent day during the Session, under the charge of the "*Reception Committee.*" Any member desiring to anticipate this Registration, can apply by letter to the Secretary General and forward his dues, with his address in full, when a receipt will be returned.

The dues of Membership for residents of the United States will be Ten dollars ($10.00). There will be no dues for members residing in other countries. Each member will be entitled to receive a copy of the "*Transactions*" of the Congress, when published by the Executive Committee.

The General Sessions of the Congress will be devoted to the transaction of business and Addresses and Communications of general scientific interest, by members appointed by the Executive Committee. A printed "Programme" of the Sessions will be presented to each member on registering. A printed "Order of Business" for each day will also be issued.

The work of the various Sections will be directed by the President of the Section, and the order will be published in a daily Programme for each Section. Questions and topics that have been agreed on for discussion in the Sections, shall be introduced by members previously designated by the titular officers of each Section. Members who shall have been appointed to open discussions, shall present to the Secretaries of the Section, in advance, statements of the conclusions which they have formed as a basis for the debate.

Brief abstracts of Papers to be read in the Sections shall be forwarded to the Secretaries of the proper Section on or before *April 30, 1887*. These abstracts shall be treated as confidential communications, and shall not be published before the meeting of the Congress. Papers relating to topics not included in the list of subjects proposed by the Officers of the Sections, may be accepted after April 30, 1887, and any member wishing to introduce a topic not on the regular lists of subjects for discussion, shall give notice of the same to the Secretary-General, at least twenty-one days before the opening of the Congress. The titular officers of each Section shall decide as to the acceptance of such proposed communications, and the time for their presentation. No communication shall be received which has been already published or read before a Society.

The official languages of the Congress shall be English, French and German. Each paper or address shall be printed in the "*Transactions*" in the language in which it was presented. Preliminary abstracts of papers and addresses shall also be printed in the language in which each is to be delivered. All discussions shall be printed in English.

The officers of the Congress and the officers of the Sections, including all foreign officers, will be nominated to the Congress by the Executive Committee, at the opening of the first session. A partial list of the officers to be nominated (except the members of Council of the different Sections, the list of whom is at present imperfect), is offered herewith.

The Executive Committee cordially invites members of the regular medical profession, and men eminent in the sciences collateral to medicine, in all countries, to participate, in person or by papers, in the work of this great humanitarian assembly. Communications relating to appointments for papers to be read in the Congress should be addressed to Dr. John B. Hamilton, Secretary-General of the Ninth International Medical Congress, Washington, District of Columbia. All questions or communications connected with the business of the Executive Committee should be addressed to Dr. Henry H. Smith, Chairman of the Executive Committee of the Ninth International Medical Congress, Philadelphia, Pennsylvania. Gentlemen named in any position in the Congress are requested to notify the Chairman of the Executive Committee, as soon as practicable, of any error in the name, title or address in this circular.

Ladies in attendance with members of the Congress, and those invited by the "Reception Committee," may attend the General Sessions of the Congress when introduced by a member. They will also be invited to attend the Social Receptions. The Executive Committee reserves the right to invite distinguished persons to any or all the meetings of the Congress. The attendance of Medical Students and others interested in the work of the various Sections or in the general addresses delivered in the Congress, will be permitted, on the recommendation of the Secretary-General or the officers of a Section, on their taking out from the Registration Committee a general ticket of admission, fee one dollar ($1.00); but such persons cannot take part in the proceedings.

All communications and questions relating to the special business of any Section, must be addressed to the President or one of the Secretaries of that Section. As many details of the Congress and numerous appointments of officers are yet to be completed, other circulars will be issued from time to time, as circumstances may demand.

The following is the list, as at present completed :

EXECUTIVE COMMITTEE OF THE CONGRESS.—Henry H. Smith, M.D., LL.D., *Chairman;* N. S. Davis, M.D., LL.D.; John B. Hamilton, M. D.; E. S. F. Arnold, M.D.; Richard J. Dunglison, M.D., *Secretary;* Abram B. Arnold, M.D.; William T. Briggs, M.D.; DeLaskie Miller, M.D., Ph.D.; James F. Harrison, M.D.; F. H. Terrill, M.D.; William H. Pancoast, M.D.; John H. Callender, M.D.; Alonzo B. Palmer, M.D., LL.D.; J. Lewis Smith, M.D.; E. Williams, M.D.; S. J. Jones, M.D., LL.D.; William H. Daly, M.D.; A. R. Robinson, M.D.; Joseph Jones, M.D.; Albert L. Gihon, M.D.; John P. Gray, M.D., LL.D.; Jonathan Taft, M.D.; Frederick S. Dennis, M.D.; A. Y. P. Garnett, M. D.

PRESIDENTS OF SECTIONS.

*General Medicine.*—Abram B. Arnold, M.D., Professor of Clinical Medicine, College of Physicians and Surgeons, Baltimore, Maryland.

*General Surgery.*—William T. Briggs, M.D., Professor of Surgery, University of Nashville, Nashville, Tennessee.

*Military and Naval Surgery and Medicine.*—Henry H. Smith, M.D., LL.D., Emeritus Professor of Surgery, University of Pennsylvania, and formerly Surgeon-General of Pennsylvania, Philadelphia, Pennsylvania.

*Obstetrics.*—DeLaskie Miller, M.D., Ph.D., Professor of Obstetrics, Rush Medical College, Chicago, Illinois.

*Gynæcology.*—James F. Harrison, M. D., Professor of Obstetrics, etc., University of Virginia, Charlottesville, Virginia.

*Therapeutics and Materia Medica.*—F. H. Terrill, M.D., Professor of Therapeutics, University of California, San Francisco, California.

*Anatomy.*—William H. Pancoast, M.D., Professor of General, Descriptive and Surgical Anatomy, Medico-Chirurgical College, Philadelphia, Pennsylvania.

*Physiology.*—John H. Callender, M. D., Professor of Physiology and Psycology, University of Nashville, Nashville, Tennnessee.

*Pathology.*—Alonzo B. Palmer, M. D., LL. D., Professor of Pathology, University of Michigan, Ann Arbor, Michigan.

*Diseases of Children.*—J. Lewis Smith, M. D., Professor of the Diseases of Children, Bellevue Hospital Medical College, New York, New York.

*Ophthalmology.*—E. Williams, M. D., Professor of Ophthalmology, Miami Medical College, Cincinnati, Ohio.

*Otology.*—S. J. Jones, M. D., LL. D., Professor of Ophthalmology and Otology, Chicago Medical College, Chicago, Illinois.

*Laryngology.*—William H. Daly, M. D., Pittsburgh, Pennsylvania.

*Dermatology and Syphilis.*—A. R. Robinson, M. D., Professor of Dermatology, New York Polyclinic, and of Histology and Pathological Anatomy, Woman's Medical College of the New York Infirmary, New York, New York.

*Public and International Hygiene.*—Joseph Jones, M. D., Professor of

Chemistry and Clinical Medicine, Tulane University; ex-President Board of Health, New Orleans, Louisiana.

*Medical Climatology and Demography.*—Albert L. Gihon, M. D., Medical Director United States Navy.

*Psychological Medicine and Nervous Diseases.*—John P. Gray, M. D., LL. D., Professor of Psychological Medicine and Medical Jurisprudence, Bellevue Hospital Medical College, Utica, New York.

*Dental and Oral Surgery.*—Jonathan Taft, M. D., Professor of Dental and Oral Surgery, University of Michigan, Ann Arbor, Michigan, Cincinnati, Ohio.

VICE-PRESIDENTS OF SECTIONS.

*General Medicine.*—W. W. Cleaver, M. D., Kentucky; J. A. Octerloney, M. D., Kentucky; P. G. Robinson, M. D., Missouri; Thos. F. Rochester, M. D., New York; Preston B. Scott, M. D., Kentucky.

*General Surgery.*—Prof. Filanus, Holland; Moses Gunn, M. D., Illinois; J. W. Hamilton, M. D., Ohio; W. H. Hingston, M. D. Canada; Jas. M. Holloway, M. D., Kentucky; J. C. Hutchison, M. D., New York; N. S. Lincoln, M. D., District of Columbia; Donald MacLean, M. D., Michigan; Donald Macrea, M. D., Iowa; M. Storrs, M. D., Connecticut.

*Military and Naval Surgery and Medicine.*—C. J. Cleborne, M. D., United States Navy; E. H. Gregory, M. D., Missouri; W. T. Hord, M. D., Medical Director United States Navy; Frederick Hyde, M. D., New York; G. L. Porter, M. D., Connecticut; Wm. E. Taylor, M. D., Medical Inspector United States Navy; Edward Warren-Bey, M. D., France; B. A. Watson, M. D., New Jersey.

*Obstetrics.*—Gustav Braun, M. D., Austria; P. Budin, M. D., France; A. L. Galabin, M. D., England; John Goodman, M. D., Kentucky; W. M. Knapp, M. D., Nebraska; R. Lowry Sibbet, M. D., Pennsylvania; Isaac E. Taylor, M. D., New York.

*Gynæcology.*—N. Bozeman, M. D., New York; Henry O. Marcy, M. D., Massachusetts; T. A. Reamy, M. D., Ohio; H. R. Storer, M. D., Rhode Island.

*Therapeutics and Materia Medica.*—Henry M. Field, M. D., New Hampshire; Albert Frické, M. D., Pennsylvania; George Gray, M. D., Ireland.

*Anatomy.*—C. W. Kelly, M. D., Kentucky; Samuel Logan, M. D., Louisiana.

*Physiology.*—

*Pathology*—Andrew Fleming, M. D., Pennsylvania; J. B. Johnson, M. D., Missouri; Henry F. Lyster, M. D., Michigan.

*Diseases of Children.*—William B. Atkinson, M. D., Pennsylvania; William G. Booker, M. D., Maryland; William H. Day, M. D., England; Dr. Cadet de Gassicourt, France; Dr. J. Grancher, France; Dr. Edward Henoch, Prussia; Adoniram B. Judson, M. D., New York; J. P. Oliver, M. D., Massachusetts; Eustace Smith, M. D., England; Charles West, M. D., England; Joseph E. Winters, M. D., New York.

*Ophthalmology.*—A. W. Calhoun, M. D., Georgia; J. J. Chisholm, M. D., Maryland; P. D. Keyser, M. D., Pennsylvania; Dudley S. Reynolds, M. D., Kentucky.

*Otology.*—Professor Johann Schnitzler, Vienna, Austria.

*Laryngology.*—M. F. Coomes, M. D., Kentucky; J. H. Hartman, M. D., Maryland; J. O. Roe, M. D., New York; E. L. Shurley, M. D., Michigan, G. V. Woolen, M. D., Indiana.

*Dermatology and Syphilis.*—James M. Keller, M. D., Arkansas; John V. Shoemaker, M. D., Pennsylvania; George Thin, M. D., London, England.

*Public and International Hygiene.*—A. Nelson Bell, M.D., New York; John Berrien Lindsley, M.D., Tennessee; J. N. McCormack, M.D., Kentucky; J. F. Y. Paine, M.D., Texas; Benjamin W. Richardson, M.D., England; John Simon, M.D., England; J. W. Thudicum, M.D., England.

*Medical Climatology and Demogiaphy.*—Dr. A. Chervin, Paris, France; Traill Green, M.D., Pennsylvania; John H. Hollister, M.D., Illinois.

*Psychological Medicine.*—Julius Althaus, M.D., England; R. H. Chase, M.D., Pennsylvania; Eugene Grissom, M.D., North Carolina; John C. Hall, M.D., Pennsylvania; P. A. Hooper, M.D., Arkansas; J. S. Jewell, M.D.; Illinois; S. S. Schultz, M.D., Pennsylvania.

*Dental and Oral Surgery.*—W. W. Allport, M. D., Illinois; S. W. Dennis, M.D., California; C. D. Ford, M.D., Michigan; H. L. McKellops, M.D., Missouri; A. T. Metcalf, M.D., Michigan; W. H. Morgan, M.D., Tennessee; A. L. Northrop, M.D., New York; L. D. Shepard, M.D., Massachusetts.

SECRETARIES.

*General Medicine.*—J. W. Chambers, M. D., Baltimore, Maryland.

*General Surgery.*—Dudley P. Allen, M. D., Cleveland, Ohio; Carl Mayde, M. D., Germany; J. R. Weist, M. D., Richmond, Indiana; A. H. Wilson, M. D., South Boston, Massachusetts.

*Military and Naval Surgery and Medicine.*—J. McF. Gaston, M. D., Atlanta, Georgia; E. A. Wood, M. D., Pittsburg, Pennsylvania.

*Obstetrics.*—A. Charpentier, M.D., Paris, France; T. Felsenreich, M.D., Vienna, Austria; W. W. Jaggard, M. D., Chicago, Illinois; John Williams, M. D., London, England.

*Gynæcology.*—Ernest W. Cushing, M. D., Boston, Massachusetts.

*Therapeutics.*—Frank Woodbury, M. D., Philadelphia, Pennsylvania.

*Anatomy.*—Henry Morris, M.D., Philadelphia, Pennsylvania.

*Physiology.*—R. W. Bishop, M. D., Chicago, Illinois.

*Pathology.*—H. M. Biggs, M. D., New York, New York; I. N. Himes, M. D., Cleveland, Ohio.

*Diseases of Children.*—Dillon Brown, M. D., New York.

*Ophthalmology.*—S. C. Ayres, M. D., Cincinnati, Ohio.

*Otology.*—S. O. Richey, M. D., Washington, D. C.

*Laryngology.*—William Porter, M. D., St. Louis, Missouri; Dr. Hermann Krause, Berlin, Prussia.

*Dermatology.*—W. T. Corlett, M. D., Cleveland, Ohio; F. E. Daniel, M. D., Austin, Texas.

*Public and International Hygiene.*—George H. Rohé, M. D., Baltimore, Maryland; Walter Wyman, M D., U. S. Marine Hospital Service, New York, New York.

*Climatology and Demography.*—Charles Denison, M. D., Denver, Colorado; James F. Todd, M. D., Chicago, Illinois.

*Psychological Medicine.*—E. D. Ferguson, M. D., Troy, New York ; E. Landolt, M. D. (Berlin, Prussia), Paris, France.

*Dental and Oral Surgery.*—Edward A. Bogue, M. D., New York, New York ; S. F. Rehwinkle, M. D., Chillicothe, Ohio.

COMMITTEE OF ARRANGEMENTS, Washington, D. C.

A. Y. P. Garnett, M. D., Chairman.

J. M. Toner, M. D., Vice-Chairman.

C. H. A. Kleinschmidt, M. D., Secretary.

D. C. Patterson, M. D., Treasurer.

*Executive Committee.*—Drs. A. Y. P. Garnett, J. M. Toner, N. S. Lincoln, C. H. A. Kleinschmidt, Surgeon-General F. M. Gunnell, M. D., U. S. Navy; Surgeon-General Robert Murray, M. D., U. S. Army; Supervising Surgeon-General J. B. Hamilton, M. D., U. S. Marine Hospital Service; Chief Medical Purveyor, J. H. Baxter, M. D., U. S. Army.

*Committee on Congressional Legislation.*—Dr. A. Y. P. Garnett, Chairman.

*Committee on Finance.*—Dr. G. L. Magruder, Chairman.

*Committee on Printing.*—D. J. B. Hamilton, Chairman.

*Committee on Reception.*—Dr. J. M. Toner, Chairman.

*Committee on Entertainments.*—Dr. N. S. Lincoln, Chairman.

*Committee on Transportation.*—Dr. J. W. H. Lovejoy, Chairman.

*Committee on Place of Meeting for Congress and Sections.*—Dr. D. C. Patterson, Chairman.

*By Order of the Executive Committee of the Congress.*
HENRY H. SMITH, M. D., Chairman.
RICHARD J. DUNGLISON, M. D., Secretary.

